# The Pathophysiological Hypothesis of Kidney Damage during Intra-Abdominal Hypertension

**DOI:** 10.3389/fphys.2016.00055

**Published:** 2016-02-23

**Authors:** Gianluca Villa, Sara Samoni, Silvia De Rosa, Claudio Ronco

**Affiliations:** ^1^Department of Health Science, Section of Anaesthesiology and Intensive Care, University of FlorenceFlorence, Italy; ^2^Department of Anaesthesiology and Intensive Care, Azienda Ospedaliero Universitaria CareggiFlorence, Italy; ^3^Department of Nephrology, Dialysis and Transplantation, International Renal Research Institute, San Bortolo HospitalVicenza, Italy; ^4^Institute of Life Sciences, Sant'Anna School of Advanced StudiesPisa, Italy; ^5^Department of Anaesthesiology and Intensive Care, A. Gemelli University Hospital, Catholic University of the Sacred HeartRome, Italy

**Keywords:** acute kidney injury, abdominal pressure, biomarkers of AKI, renal blood flow, glomerular filtration

The increase in intra-abdominal pressure (IAP) above specific levels (i.e., intra-abdominal hypertension, IAH) may lead to organ dysfunction in abdominal and extra-abdominal systems (Kirkpatrick and Roberts, 2013). Possible etiologies or risk factors for IAH development comprehend diminished abdominal compliance, increased intraluminal or intra-abdominal contents and capillary leak/fluid resuscitation (Kirkpatrick and Roberts, 2013). In this conditions, formally known as abdominal compartment syndrome, acute kidney injury (AKI) frequently develops and further worsens the patients outcome (Dalfino et al., 2008).

Pathophysiological mechanisms leading to AKI during IAH are not completely known; nevertheless, evidence from the literature recognize the decrease in renal perfusion as the main factor responsible for development of AKI in this condition (De Waele et al., 2011). In particular, renal hypoperfusion might occur during an acute or progressive increase in IAP, mainly due to the reduction of both arterial inflow and venous outflow, leading to glomerular hemodynamic alterations.

Beyond the subsequent activation of neuro-hormonal pathways (e.g., noradrenergic response and Renin-Angiotensin-Aldosteron system), the intrarenal hemodynamic alteration may be itself the responsible for an acute decrease of glomerular filtration gradient (FG; De Waele et al., 2011). The FG reflects the balance among hydrostatic and oncotic forces that support the ultrafiltration through the glomerular barrier. During IAH, the decrease of glomerular hydrostatic pressure (due to hypoperfusion) and the increase of Bowman's space hydrostatic pressure (due to IAH) may lead to acute reduction in FG (De Waele et al., 2011). Data from literature confirm an inverse correlation between IAP and FG (Harman et al., 1982).

Physiologically, an acute increase in IAP narrows renal arteries and veins, reduces renal blood flow, leading to the activation of autoregulatory mechanisms. These cause a vasodilation of afferent arterioles, ensuring glomerular filtration also during the early stage of acute increase in IAP (Just, 2007). Probably, the activation of these mechanisms may determine an acute increase in glomerular filtration during stressful events and we hypothesized that it might be related to the patient's renal functional reserve. Moreover, the same IAP value may produce different levels of decreased renal function related to different levels of myogenic response influencing the efficiency of autoregulatory mechanisms.

According to experimental data showed by Harman et al., Figure 1 represents the correlation between current renal function (*x axis*) and IAP (*y-right axis*) (Harman et al., 1982). In patients with effective myogenic response (patient n°1, dashed line), an acute increase in IAP is associated to a slight decrease in renal function. Whereas, in patients with a compromised myogenic response (patient n°2, solid line), and lower renal functional reserve, an acute increase in IAP is associated with a strong reduction in renal function.

**Figure 1:**
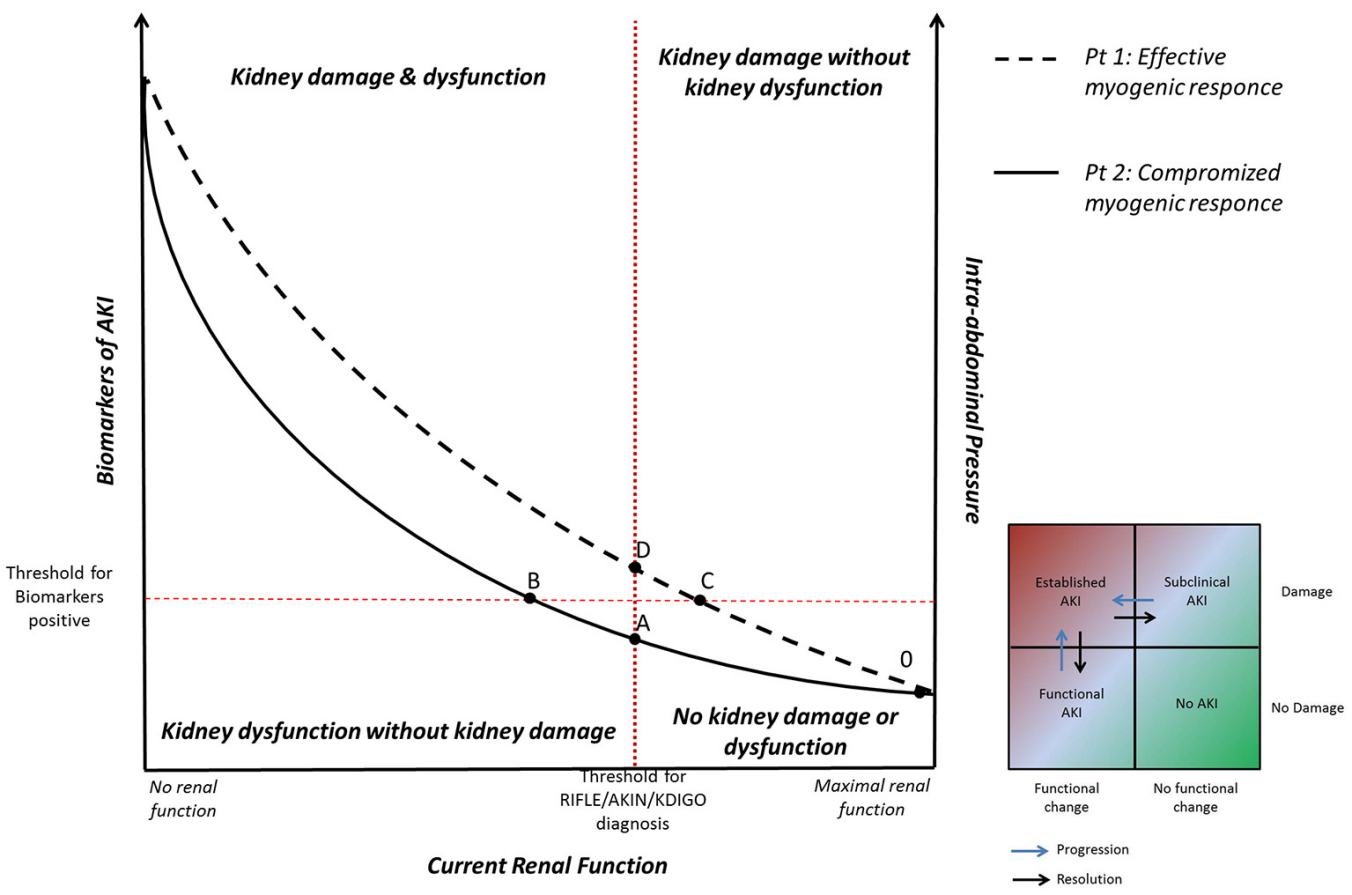
**Correlation between current renal function, intra-abdominal pressure (IAP), and biomarkers of acute kidney injury (AKI)**. Patient n° 1 (dashed line): in presence of effective myogenic response, an acute increase in IAP is associated to a slight decrease in renal function (tract 0–C). A further increase of IAP may lead to biomarkers increase (subclinical AKI, tract C–D). When IAP overcomes the intrarenal autoregulation, glomerular hypoperfusion occurs and a picture of clinical AKI becomes manifest (above point D). Patient n° 2 (solid line): in presence of compromised myogenic response, an acute increase in IAP is associated with a strong reduction in renal function until the development of clinical functional AKI (tract A–B). If IAP further increases, the inflammatory and ischemic insults may lead to the kidney parenchymal damage detectable by biomarkers (above point B).

Although the hemodynamic issue is certainly quintessential to explain the pathophysiology of AKI during IAH, other mechanisms may further affect the kidney function (e.g., the direct parenchyma compression or the inflammatory damage; Doty et al., 2000; Kösüm et al., 2013).

Beyond the etiological conditions leading to the acute increase in IAP, the IAH itself may induce systemic inflammation (Rezende-Neto et al., 2002). Indeed, it is well known as systemic inflammation can widely sustain AKI through circulating biochemical factors inducing apoptotic/necrotic damages to the renal parenchyma (Honore et al., 2011). Furthermore, also metabolic alterations induced locally may be recognized in the kidney during IAH. In particular, during IAP elevation a widely range of genes are up- and down-regulated in the kidney, leading to a dynamic and constantly changing metabolic response (Edil et al., 2003). In experimental models of IAH, high levels of locally-produced inflammatory mediators (e.g., TNF-a or IL-6) have been demonstrated in the kidney during the IAP elevation as well as their association with histopathological and cytoarchitectural alterations (Akbulut et al., 2010; Kösüm et al., 2013).

The susceptibility to kidney damage due to hemodynamic or biological insults during “IAH exposure” might be theoretical detectable through the use of biomarkers of AKI (Li et al., 2014). Several biomarkers have been proposed to identify the kidney damage during clinical scenarios at risk for AKI, for example the perioperative urinary liver-type fatty-acid-binding protein during endovascular abdominal repair (Obata et al., 2016). Although most of literature provides information on specific molecules, such as neutrophil gelatinase-associated lipocalin or Kidney injury molecule-1, biomarkers of cell-cycle arrest have been recently identified as the most sensitive and specific biomarkers for AKI in most clinical settings (Kashani et al., 2013). According to ADQI classifications (McCullough et al., 2013), biomarkers of AKI might identify the parenchyma kidney damage occurred after a metabolic insult, whereas the clinical classifications based on urinary output or serum creatinine (aimed to quantify the glomerular filtration rate) might identify the kidney dysfunction (Ronco et al., 2012). As demonstrated in literature, clinical AKI is widely correlated with an increased patients' mortality; in these conditions the use of biomarkers of kidney damage might inform about severity, prognosis, and recovery from AKI (Endre et al., 2011). Nevertheless, also conditions characterized by an increase of biomarkers of kidney damage, but in which clinical scoring systems fail to identify a kidney dysfunction (i.e., “subclinical AKI”) are associated to patients' mortality (Ronco et al., 2012).

The identification of the pathophysiological mechanisms for AKI, as well as the quantification of specific patient's responses to pathological stimuli (as myogenic activation and renal functional reserve) and the evaluation of the kidney damage/dysfunction, should be achieved during IAH-induced AKI. This may allow a personalized treatment for that specific patient and a target-directed therapy for AKI (Joannidis et al., 2010) even during IAH.

In particular, in patient n°2 (with low myogenic response) renal function rapidly falls during IAP elevation until the development of clinical AKI (from point A). In this situation, the ineffective response of the patient to the reduced glomerular perfusion might produce a clinical “functional” AKI even if the parenchymal kidney damage (biological or ischemic) does not actually occurred (tract A–B). In this phase the optimization of cardiac output and/or volume replacement might increase the renal perfusion restoring glomerular function. If IAP further increases, the biological inflammatory insult, as well as the ischemic insult deriving from hypoperfusion, may lead to the kidney parenchymal damage detectable by biomarkers (above point B).

On the other hand, in patient n°1 (with an effective myogenic response) renal function slightly decreases during the IAP elevation (from point 0 to C). The patient's effective intrarenal autoregulation allows him to maintain the glomerular filtration pressure in this early phase, avoiding the “functional” AKI (deriving from hypoperfusion or hypovolemia). However, the progression of IAH and the subsequent biological inflammatory insult may lead to kidney damage clinically detectable through the biomarkers increase (point C). In this specific condition, the reduction of renal function occurs in a picture of subclinical AKI (from point C to D), in which the patient's parenchymal damage is associated to a normal glomerular function sustained by intrarenal autoregulation. If IAH progresses, the IAP overcomes the intrarenal autoregulation, glomerular hypoperfusion occurs meanwhile the biological insult progresses and a picture of clinical AKI becomes manifest (above point D).

In conclusion, although pathophysiological mechanisms responsible to AKI during IAH are not completely understood, the decrease in renal perfusion is one of the most important causative factor (De Waele et al., 2011). The acute increase of intra-abdominal pressure reduces the renal blood flow and triggers the autoregulatory mechanisms, acutely rising glomerular filtration. The integrity of myogenic response might be related to the patient's capability to maintain an adequate glomerular filtration rate during stressful conditions (e.g., metabolic load or hemodynamic insult leading to kidney hypoperfusion). Other etiological stimuli such as inflammatory end/or toxic exposures may also induce kidney impairment and/or kidney dysfunction during IAH, thus leading to clinical or subclinical AKI. In a comprehensive approach to the kidney function during IAH, the evaluation of myogenic response with the clinical and biochemical parameters of AKI may have a role to personalizing the treatment for each specific patient.

## Author contributions

GV, SS, and SD have substantially contributed to the conception of the work, drafting the work or revising it critically for important intellectual content. CR has finally approved the version to be published.

### Conflict of interest statement

The authors declare that the research was conducted in the absence of any commercial or financial relationships that could be construed as a potential conflict of interest.

## References

[B1] Akbulut G. Altindis M. Aktepe F. Serteser M. Dilek O. N. (2010). Renal cytokine and histopathologic changes following acutely increased intra-abdominal pressure: an animal study. Ulus. Travma Acil. Cerrahi. Derg. 16, 103–107. 20517761

[B2] Dalfino L. Tullo L. Donadio I. Malcangi V. Brienza N. (2008). Intra-abdominal hypertension and acute renal failure in critically ill patients. Intensive Care Med. 34, 707–713. 10.1007/s00134-007-0969-4 18157662

[B3] De Waele J. J. De Laet I. Kirkpatrick A. W. Hoste E. (2011). Intra-abdominal hypertension and abdominal compartment syndrome. Am. J. Kidney Dis. 57, 159–169. 10.1053/j.ajkd.2010.08.034 21184922

[B4] Doty J. M. Saggi B. H. Blocher C. R. Fakhry I. Gehr T. Sica D.. (2000). Effects of increased renal parenchymal pressure on renal function. J. Trauma 48, 874–877. 10.1097/00005373-200005000-00010 10823530

[B5] Edil B. H. Tuggle D. W. Puffinbarger N. K. Mantor P. C. Palmer B. W. Knutson Z. A. (2003). The impact of intra-abdominal hypertension on gene expression in the kidney. J. Trauma 55, 857–859. 10.1097/01.TA.0000093394.22151.7A 14608156

[B6] Endre Z. H. Pickering J. W. Walker R. J. (2011). Clearance and beyond: the complementary roles of GFR measurement and injury biomarkers in acute kidney injury (AKI). Am. J. Physiol. Renal Physiol. 301, F697–F707. 10.1152/ajprenal.00448.2010 21753074

[B7] Harman P. K. Kron I. L. McLachlan H. D. Freedlender A. E. Nolan S. P. (1982). Elevated intra-abdominal pressure and renal function. Ann. Surg. 196, 594–597. 10.1097/00000658-198211000-00015 7125746 PMC1352794

[B8] Honore P. M. Jacobs R. Joannes-Boyau O. De Regt J. Boer W. De Waele E.. (2011). Septic AKI in ICU patients. diagnosis, pathophysiology, and treatment type, dosing, and timing: a comprehensive review of recent and future developments. Ann. Intensive Care 1:32. 10.1186/2110-5820-1-32 21906387 PMC3224527

[B9] Joannidis M. Druml W. Forni L. G. Groeneveld A. B. J. Honore P. Oudemans-Van Straaten H. M.. (2010). Prevention of acute kidney injury and protection of renal function in the intensive care unit: expert opinion of the working group for nephrology, ESICM. Intensive Care Med. 36, 392–411. 10.1007/s00134-009-1678-y 19921152

[B10] Just A. (2007). Mechanisms of renal blood flow autoregulation: dynamics and contributions. Am. J. Physiol. Regul. Integr. Comp. Physiol. 292, R1–R17. 10.1152/ajpregu.00332.2006 16990493

[B11] Kashani K. Al-khafaji A. Ardiles T. Artigas A. Bagshaw S. M. Bell M.. (2013). Discovery and validation of cell cycle arrest biomarkers in human acute kidney injury. Crit. Care 17:R25. 10.1186/cc12503 23388612 PMC4057242

[B12] Kirkpatrick A. Roberts D. (2013). Intra-abdominal hypertension and the abdominal compartment syndrome: updated consensus definitions and clinical practice guidelines from the world. Intensive Care Med. 39, 1190–206. 10.1007/s00134-013-2906-z 23673399 PMC3680657

[B13] Kösüm A. Borazan E. Maralcan G. Aytekin A. (2013). Biochemical and histopathological changes of intra-abdominal hypertension on the kidneys: experimental study in rats. Turkish J. Surg. 29, 49–53. 10.5152/UCD.2013.39 25931845 PMC4379841

[B14] Li W. Cao Z. Xia Z. Meng Q. Yu W. Yao X.. (2014). Acute kidney injury induced by various pneumoperitoneum pressures in a rabbit model of mild and severe hydronephrosis. Urol. Int. 94, 225–233. 10.1159/000362845 25196500

[B15] McCullough P. A. P. Shaw A. A. D. Haase M. Bouchard J. Waikar S. S. S. Siew E. D.. (2013). Diagnosis of acute kidney injury using functional and injury biomarkers: workgroup statements from the tenth acute dialysis quality initiative consensus conference. Contrib. Nephrol. 182, 13–29. 10.1159/000349963 23689653

[B16] Obata Y. Kamijo-Ikemori A. Ichikawa D. Sugaya T. Kimura K. Shibagaki Y.. (2016). Clinical usefulness of urinary liver-type fatty-acid-binding protein as a perioperative marker of acute kidney injury in patients undergoing endovascular or open-abdominal aortic aneurysm repair. J. Anesth. 30, 89–99. 10.1007/s00540-015-2095-8 26585768 PMC4750552

[B17] Rezende-Neto J. B. Moore E. E. Melo de Andrade M. V. Teixeira M. M. Lisboa F. A. Arantes R. M. E.. (2002). Systemic inflammatory response secondary to abdominal compartment syndrome: stage for multiple organ failure. J. Trauma 53, 1121–1128. 10.1097/00005373-200212000-00015 12478038

[B18] Ronco C. Kellum J. A. Haase M. (2012). Subclinical AKI is still AKI. Crit. Care 16:313. 10.1186/cc11240 22721504 PMC3580601

